# Enhanced Three Layer Hybrid Clustering Mechanism for Energy Efficient Routing in IoT

**DOI:** 10.3390/s19040829

**Published:** 2019-02-18

**Authors:** Muhammad Faizan Ullah, Junaid Imtiaz, Khawaja Qasim Maqbool

**Affiliations:** Department of Electrical Engineering, Bahria University, Islamabad 44000, Pakistan; 01-244171-015@student.bahria.edu.pk (M.F.U.); qasim@Bahria.edu.pk (K.Q.M.)

**Keywords:** wireless sensor network, IoT, clustering, energy efficiency

## Abstract

Recently, different routing techniques were proposed for three layer clustering topology in Wireless Sensor Network (WSN) which outperform the basic two layer clustering hierarchy. The problem that remains in these approaches is the heavy control packet exchange between nodes after every round in order to choose efficient lower layer heads. Among these techniques is Hybrid Hierarchical Clustering Approach (HHCA), in which a distributed approach is proposed. According to HHCA, the upper layer heads are centrally selected by base station, while sensor nodes only have to select lower layer heads distributively. In this paper, enhanced three layer hybrid clustering mechanism is proposed that limits the exchange of control packets between nodes after every round for lower layer head selection. The energy of nodes are divided into levels upon which it is decided when nodes of a cluster need to enter into new cluster head selection phase. The proposed mechanism helps to limit control packet exchange between nodes to a large extent, at the same time keeping energy consumption between nodes balanced. Moreover, it is focused that higher layer heads are selected by base station in a manner that reduces backward transmission in the network as much as possible. Simulation results show that nodes in the proposed mechanism stay alive for a longer time as compared to other approaches, and it outperforms HHCA technique in network lifetime based on Half of the Nodes Alive (HNA) by 18 percent.

## 1. Introduction

Wireless Sensor Network (WSN), which is also called ear and eyes of Internet of Things (IoT), becomes an important research topic due its increasing role and usage in many applications. WSN as a connecting bridge between real and digital worlds is a fast growing field of IoT that provides the required data from the field accurately with low cost. The common vision of different IoT wireless systems is usually associated with same concept, use of networked embedded devices to achieve intelligent monitoring and management. Devices are interconnected to transmit useful measurement information and control instructions via distributed sensor networks.

WSN is widely used in both delay tolerant and real time applications. Some well known usages of WSNs are: Smart cities and smart homes applications, environment and wildlife monitoring, security and military related applications, disaster management and rescue applications, and others [1]. The application area of WSN is enlarging day by day, with rapid development in the fields of low power consuming wireless communication [2] and digital electronics [1].

WSN consists of one or more sink nodes, and many sensor nodes. Usually a large number of homogeneous nodes are deployed randomly in the field of interest to collect required attributes and transmit them towards the sink node, which in turn forwards these data packets to the nearest station.

A sensor node is typically a tiny electronic device equipped with: Low cost micro controller with very low power consumption, one or more sensors that gather data of interest from the surroundings, limited battery which is assumed to neither be replaced nor recharged as the power source, flash memory, and transceiver that uses Radio Frequency (RF) for communication with other nodes. Laser and infrared are also can be used for communication instead of RF in some WSN applications, but they are sensitive to atmospheric conditions, and also require line of sight. RF based communication is most popular in WSN applications. License free Industrial Scientific and Medical (ISM) frequencies radio bands such as 433 MHz, 915 MHz, and 2.4 GHz are mostly used in WSNs.

It is obvious that these small low cost sensor nodes are neither accurate nor powerful as compared to the expansive macro sensor nodes, but a good quality sensor network still can be made by using large number of these nodes together. Normally, these nodes are deployed randomly in remote areas where deployment of large expansive nodes is not possible due to high cost or harsh environment.

Sensor nodes have limited source of power and their batteries in most cases cannot be recharged or replaced due to many reasons such as: Large number of deployed sensor nodes, random deployment of sensor nodes, remote location of the sensing region, and harsh environment. Therefore, energy remains the main issue and there is always a need for algorithms and mechanisms that reduce network energy consumption and balance consumption of power between all deployed sensor nodes, in order to prolong the network life time. Unlike sensor nodes, the sink node is assumed to have no power constraints and its main task is to gather sensed information from the deployed sensor nodes and forward them to the nearest station.

A sensor node mainly use energy in two conditions. The first condition is when a sensor node performs tasks such as transmitting data packets, receiving data packets, processing requests; which are called useful tasks. While operations such as transmission and reception of control packets with neighboring nodes for upper or lower layer head selection or for choosing better forwarder, retransmissions, overhearing, processing redundant packets, and similar other operations come under second condition, which is considered a waste of energy.

Network lifetime is considered a major factor in performance evaluation of routing protocols for WSN when sensor nodes have limited power source. Many techniques have been proposed in the past few years to reduce energy consumption for getting longer network lifetime. One of the low energy consumption techniques in WSN is formation of clusters and routing data packets towards sinks via cluster heads. The basic Low Energy Adaptive Clustering Hierarchy (LEACH) protocol [3] is a two layer clustering technique in which Cluster Heads (CHs) are selected randomly after every round. At the beginning of every new round, nodes are assigned random number and the one with higher random value is assigned as CH at that round, giving priority to those nodes which are not selected as CH previously. Many improvements are proposed in the choice method of CH in LEACH protocol such as [4,5,6,7].

Recently, three layer clustering hierarchies are proposed for energy efficient data routing in WSNs, which have longer network lifetime as compared to basic two layer clustering techniques. HHCA protocol [8] is an adaptive three layer clustering protocol. Every node in this protocol firstly sends its location and residual energy information to the sink. The sink selects upper layer heads which are named as Grid Heads (GH). Sensor nodes using fuzzy c-means clustering approach (FCM) [9] join grid, and then exchange control packets with each other for selection of CH using the same distributive procedure as used in LEACH with taking residual energy of sensor nodes into account, too.

The three layer approach and combination of centrally selection of upper layer GHs via sink and distributive selection of lower layer CHs via sensor nodes shows better results as compared to fully distributive clustering techniques. The problem remaining in this approach is a waste of excessive amounts of energy in transmitting and receiving control packets after every complete round for assigning role of GH and CH to nodes.

**Figure 1:**
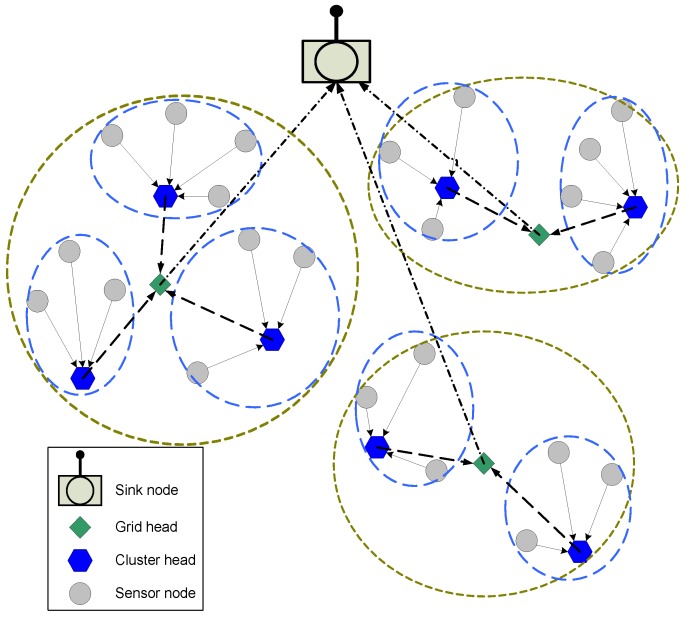
Three layer clustering topology.

In Figure 1, a typical three layer clustering topology in WSN is shown.

In this paper, an Enhanced Three Layer Hybrid Clustering Mechanism (ETLHCM) is proposed that tackles, to some extent, the issue of large wasteful operations in previous three layer clustering techniques. Energy Levels (EL) for sensor nodes are designed in this paper to limit the exchange of control packets after every round for CH selection. At the same time, EL also used to balance energy consumption between sensor nodes. Nodes that are selected as upper or lower layer heads change their role back to normal sensor node only when their residual energy drops by at least 1 EL. The proposed mechanism reduces the burden of election and selection of lower layers heads in every round, as well as reduces to a large extent the control packet exchange overhead. By reducing wasteful operations, ETLHCM shows better results in the form of long network lifetime and high number of data packets received by the sink. Details of the proposed mechanism are presented in Section 4.

Moreover, we added euclidian distance to the sink as another prioritizing parameter besides the residual energy of sensor node in fuzzification process for selection of GHs via FCM approach. Thus, sensor nodes with higher residual energy and less euclidean distance to the sink have more priority to be selected by sink as GH. This results in reducing backward transmission of data packets from lower layer to upper layer to some extent. The proposed mechanism can be used to increase the efficiency of IoT devices by reducing un necessary communication overhead.

The rest of the paper is organized as follows. Some energy efficient routing techniques and algorithms are defined in Section 2. In Section 3 issues of existing recent work in three layer routing algorithms are explained in details. Section 4 explains the proposed mechanism and simulation results are evaluated and compared with recent existing works in Section 5. The conclusion and future work are presented in Section 6.

## 2. Related Work

Hierarchical transmission in WSN is considered one of the preferred ways for energy efficient routing of data packets toward the destination. In this kind of architecture, sensor nodes are divided into different layers with different tasks.

Clustering is a technique where different sensor nodes divided into groups and sub groups, they transmit their sensed data to the CH node and they in turn forward these packets toward the sink in hierarchical fashion. LEACH protocol [3] is considered one of the basic and simple two layer cluster routing technique in WSN, in which single hop communication is used between base station and CH. In LEACH, the CH role is circulated over all nodes one by one randomly; prioritizing those nodes to be selected as CH that had not been selected before. The approach proposed in LEACH lacked efficiency in many aspects, especially in the selection process of CH among deployed homogenous sensor nodes.

The authors in [10] take two other factors in consideration before assigning CH role to any sensor node, which are the communication cost and the residual energy of sensor nodes. Enhanced Energy Efficient Adaptive Clustering (EEEAC) protocol [11] is also based on residual energy of sensor nodes and uniformly distributes the CH overload in the network between sensor nodes. While most energy efficient protocols extend network lifetime on the cost of end to end delay or other network parameters, the authors in [12] propose an energy aware protocol that maintains good end to end delay and throughput as well.

The authors in [13] prolong the network lifetime by dividing the entire network region into tiers. Sensor nodes of high amount of energy and shortest distance to the sink are selected as CHs in this protocol. In [14], an opportunistic multi path routing mechanism is proposed with aim to minimize the energy consumed due to the selection process of forwarding nodes in order to prolong the network lifetime.

A distributed algorithm called Scalable Energy Efficient Clustering Hierarchy (SEECH) is proposed in [15]. SEECH selects low degree nodes as relays while high degree nodes are selected as CHs separately using distance based algorithm.

In [16], the size of clusters are chosen to be variable. Thus, clusters with maximum distance to sink have larger size as compared to those that are nearer to sink. A sub tree mechanism is proposed in this protocol so that some parents nodes gather data packets from their neighboring sensor nodes and forward them to CH, which in turn transmits them toward the base station.

Cooperative communication improves the capacity of transmission and achieves spectral efficiency by taking advantage of the broadcast nature of communication in WSN [17]. Cooperative communication is basically developed from Multiple Input Multiple Output (MIMO) mechanisms, in which multiple sensor nodes share their resources to cooperatively reach data packets from source to destination without the need to equip each and every sensor node with multiple antennas.

Relay selection Based Cooperative Routing (RBCR) is defined in [18], which is a relay selection based cooperative technique. Decode and forward without redundancy check strategy is adopted in RBCR with selection of best paths according to the information of channel state. At the receiving node, all receiving signals are combined again to retrieve the actual sent data packet.

Recently, researchers are working on using controllers that are used in the Software Defined Network (SDN) to perform all computational task of WSN. Software Defined Wireless Sensor Network (SDWSN) play important roles in reducing energy consumption and prolonging network lifetime of WSNs. The SDN model also helps in adding many other functionalities and more intelligence to the WSN. A centralized SDWSN model is described in [19]. The main problem in fully centralized WSN or SDWSN is that it is totally dependent over controller, which makes the network less responsive in some conditions. For example, if a CH node dies, the data packets from its neighboring nodes should be re-routed, but it cannot be happen in a fully centralized mechanism until the new schedule is not updated by the central controller. On the other hand in distributive case, sensor nodes can handle re-routing locally by themselves very quickly [20]. A combination of centralized and distributive approaches has better outcomes.

In [21], the authors propose a traffic deduction algorithm that reduce the overall all data packets volume by exploring intra correlation of data packets generated by the sensor nodes. This algorithm prioritizes the unusual detected data packets and guarantee its delivery to the base station. Geographic routing is also broadly used in WSN due to its efficiency and simplicity. However, it suffers from routing holes where there is no eligible node to forward data packets further and thus data packets are stopped at the hole boundary. The authors in [22] propose a distributed hole bypassing mechanism that tackles the load imbalance as well as routing path enlargement issues.

In [23], a distributed learning automation (DLA) based algorithm is proposed to improve network lifetime by taking many routing constraints such as end to end delay and reliability into consideration in the selection process of routes for data forwarding toward the base station. A traffic minimization mechanism is proposed in [24] to reduce the data required for communication by exploring the intra correlations of the sensors data. This mechanism which is proposed to detect landslide, reduces energy consumption of WSN without degrading the detection performance of sensor nodes.

The authors in [25] define a topology control protocol in which the deployed sensor nodes learn and choose proper transmission range from the reinforcement signals of neighboring nodes. The network lifetime is increased by adopting lower transmission range for every node. The authors in [26] propose algorithm that provides certain level of protection to each sensor node with minimum energy consumption.

## 3. Framework of Proposed ETLHCM

In this section, the issues of recent work over three layer clustering routing in WSN are highlighted and the motivation for the proposed mechanism in this paper is explained.

Recently in “an improved three-layer low-energy adaptive clustering hierarchy for wireless sensor networks” [8] paper, a new Hybrid Hierarchical Clustering Approach (HHCA) is proposed for three layer clustering routing, to improve the network lifetime of WSN. According to HHCA, all deployed sensor nodes in the first step transmit their location information along with their energy status to the sink node. By applying centralized Fuzzy C-Means (FCM) clustering approach [9], every sensor node determines wether it is selected by sink as GH. In case a sensor node is selected as GH, it broadcasts a control packet announcing its selection. If the sensor node is not selected as GH, it joins the grid that is selected to it by sink via FCM approach. The second step in HHCA is that all sensor nodes exchange control packets with each other and distributively select lower layer heads which are also called cluster heads in the same way as proposed in LEACH, with the addition of taking residual energy of nodes into consideration too, giving priority to sensor nodes with higher residual energy during CH selection process. Sensor nodes that are selected as CHs broadcast control packet announce their assigned role, while other sensor nodes send join requests to nearest CH. Both the first and second steps in HHCA protocol are repeated after every round. Finally, all sensor nodes that are not selected as CH and GH transmit their data packets to CHs, and CHs in turn forward these data packets to GHs and they then forward to the sink.

It is noticed that the operations which are categorized as wasteful in Section 1 are taking place in large amounts after every round in the aforementioned protocol. The repeated exchange of control packets between sensor nodes in HHCA and other routing techniques is best for lower layer heads selection and energy balancing purpose, which results in waste of significant amounts of network energy. As stated in [27], the high power consuming operation in WSN is communication, i.e., sending and receiving packets, takes about two thirds of the total power consumption of a wireless module.

To save this excessive amount of energy that is wasted by exchanging control packets between sensor nodes, ETLHCM mechanism is proposed in this paper to limit the exchange of control packets after every round and in the mean time keeps energy consumption between sensor nodes balanced.

## 4. Network Model of ETLHCM

In this paper, first order radio model is adapted from [28]. It is used to calculate the energy that is consumed in transmitting ($E_{TX}$) and receiving ($E_{RX}$) of packets size *l* bits over distance *d* by each deployed sensor node in the network.

(1) $$\;E_{TX}=\left\{\begin{array}{c} l*\left(E_{elc}+\varepsilon_{fs}*d^{2}\right),\;d<d_{0}\\ l*\left(E_{elc}+\varepsilon_{mp}*d^{4}\right),\;d\geq d_{0} \end{array}\right.$$

Distance threshold $d_{0}$ is the normal transmission range of sensor node, $E_{elc}$ and $\varepsilon_{fs}$ are the energy dissipation to run the radio and free space model of transmitter amplifier and their values are 50 nJ/bit and 10 pJ/bit/m${}^{2}$ respectively. *l* is the data packet size and $\varepsilon_{mp}$ is the multi path model of transmitter amplifier and its value is 0.0013 pJ/bit/m${}^{4}$.

The receiving energy $E_{RX}$ can be calculated as:

(2) $$E_{RX}=l*E_{elc}$$

**Figure 2:**
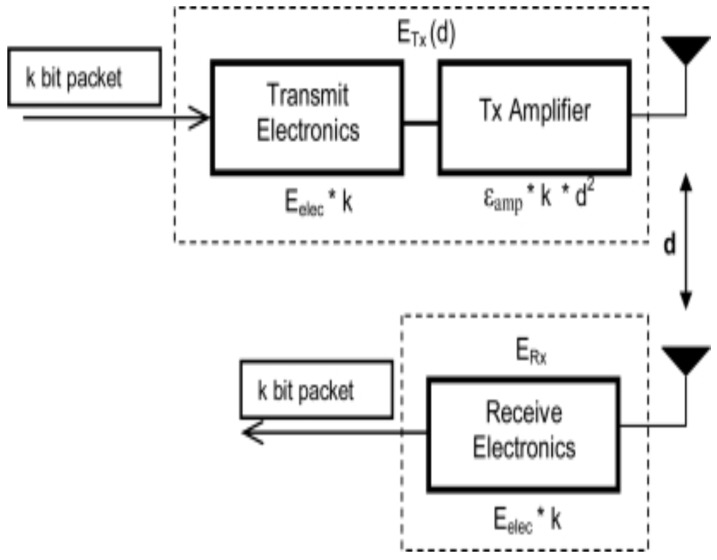
Energy consumption in transmitting and receiving data packet in first order radio model.

Figure 2 explains the aforementioned ($E_{TX}$) and ($E_{RX}$) consumption process in first order radio model [28].

It is assumed in this paper that all sensor nodes are randomly deployed in the field of fixed dimension. The deployed sensor nodes are homogenous and all have same capabilities and limited power source of same initial energy that cannot be recharged or replaced. The sink node is assumed to have no energy constraints and is deployed outside the coverage area. All sensor nodes are assumed to know about the location of the sink node and are able to find their own location information after deployment. Sensor nodes are static and do not change their locations once they are deployed. Sensor nodes determine the distances between each other based upon the received signal strength.

In the initial round, all deployed sensor nodes transmit their location and energy information hop by hop to sink. Sensor nodes join grid or upper layer in the same mechanism as explained in [9]. Nodes which are selected as grid head broadcast their assigned role, and pause the sensing role. Other sensor nodes, after joining upper layer, start the exchange of control packets between each other for cluster head or lower layer head selection. All sensor nodes share their residual energy and location information via control packet. Fuzzification process of shared parameters by sensor nodes takes place distributively. The node of higher residual energy and lower Euclidean distance to both sink and grid head is chosen to be cluster head. The euclidean distance between any two sensor nodes *a* and *b* is calculated from the following two dimension euclidean distance Equation (3).

(3) $$d\left(a,b\right)=\sqrt{{\left(x_{1}-x_{2}\right)}^{2}+{\left(y_{1}-y_{2}\right)}^{2}}$$

where $x_{1}$ and $x_{2}$ are the width dimensions, $y_{1}$ and $y_{2}$ are length dimensions of sensor nodes *a* and *b* respectively.

Nodes with CH role collect data from sensor nodes, compress it, and transmit it toward GH, which in turn forward them toward sink, while the rest of the sensor nodes sense the environment, collect data, and transmit it to their CH.

Unlike HHCA and other previous three layer routing techniques, sensor nodes are not required to repeat the cluster head selection process and exchange of control packets between each other after every round. In the proposed mechanism, the main goal is to minimize the wasteful operations in every round and save nodes energy. Equation (4) shows the objective function of the proposed mechanism.

(4) $$Min\sum_{r=1}^{r=max}WE_{consumed}\left(r\right)\forall r\in R$$

$WE_{consumed}$ is calculated using Equation (5).

(5) $$WE_{consumed}=\sum_{i=1}^{N}d_{o}\left(i\right)\times \left(E_{TX}ControlPacket+E_{RX}ControlPacket\right)$$

where $WE_{consumed}$ is the amount of energy consumed per round in the exchange of control packets between all deployed sensor nodes *N* for CHs selection. $d_{o}$ is the maximum distance between two neighboring sensor nodes. $E_{TX}ControlPacket$ and $E_{RX}ControlPacket$ are the energy consumed in transmitting and receiving control packets. Their values can be calculated using Equations (1) and (2) respectively, given that *l* is equal to the control packet size.

For achieving the objective function which is described in Equation (4), we split the total energy of sensor nodes into different equal portions called energy levels (EL). EL is calculated from Equation (6).

(6) $$EL=E_{o}/TL$$

where $E_{o}$ is the fixed initial energy of sensor node and TL is the total energy levels. The value of TL depends upon the frequency of energy consumption by a node, density of the network, and data packet size. As TL is inversely proportional to EL, so a lower value of TL will result in a higher value of EL. The reason that we cannot set very low values to TL is due to its tradeoff with balance energy consumption between all deployed sensor nodes. The value of TL in this paper is set to 10 based on different simulation experiments.

**Figure 3:**
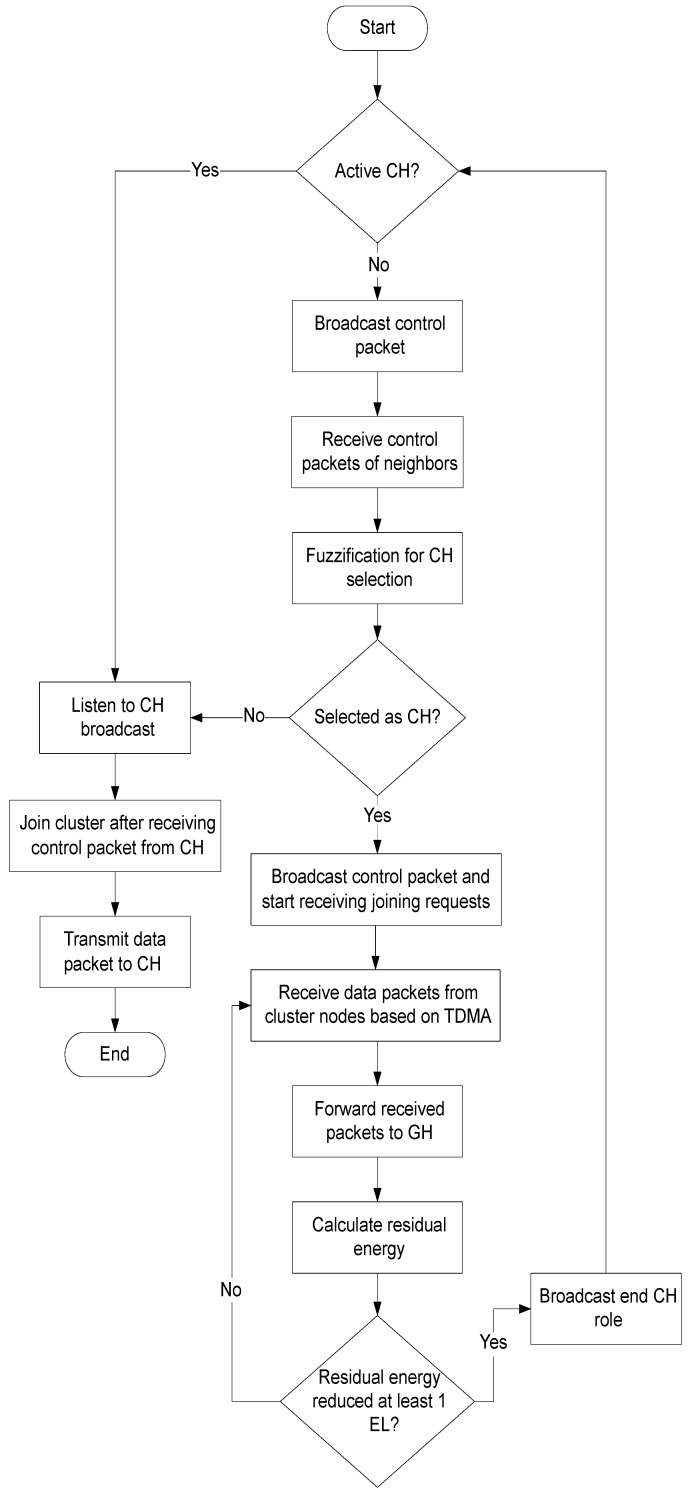
Cluster joining and cluster head (CH) selection process in the proposed mechanism.

Unless the residual energy of a sensor node that is selected as CH is not decreased by EL, it remains as CH, and no reselection process of CH take place. Whenever the CH residual energy decreased by EL, it broadcasts a control packet announcing end of its CH role. Thus new CH selection process start. The working procedure of the proposed mechanism is further explained in a flow chart which is shown in Figure 3.

## 5. Performance Evaluation

Simulation parameters are given in Table 1. Here we evaluate the simulation results of the proposed technique. Simulations are conducted using MATLAB/Simulink. One hundred nodes of the same initial energy are randomly deployed over a network of 100 m * 100 m. The Sink node is placed outside the covered area at (50 m, 175 m) in the 2D network. The main goal of the proposed technique is to prolong the network life time by avoiding retransmissions and heavy control packet exchange between sensor nodes every round.

**Figure 4:**
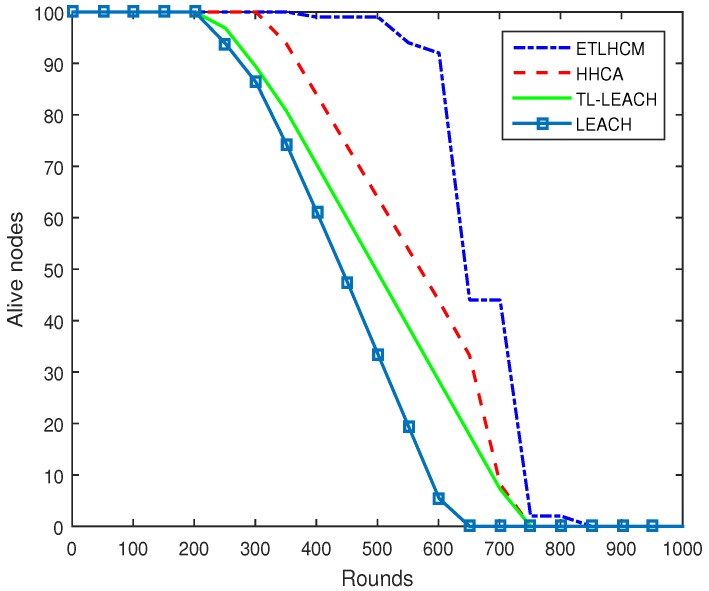
Network lifetime of proposed mechanism is increased by limiting wasteful operations.

Figure 4 shows the network lifetime of the proposed technique compared with other recent schemes. The shorter network lifetime of HHCA technique is due to the heavy control packet exchange between sensor nodes after every round for selection of efficient CH at lower layer. HHCA results are improved from the basic clustering technique LEACH and its improved version for three layer clustering routing TL-LEACH scheme, which are fully distributive clustering techniques. The main cause of low performance of LEACH is the inefficiency in procedure of CH selection. TL-LEACH tackled that problem, but since it is a fully distributive approach, it consumes more energy than a hybrid approach. While HHCA outperforms LEACH and TL-LEACH, it still consumes high energy on selection of appropriate forwarders at lower layer. In the proposed scheme, exchanging control packets between sensor nodes is limited to a large extent by defining energy levels that control in a balanced manner the rotation of CH role among the deployed sensor nodes. Results show that sensor node energy lasts for a longer time in the proposed mechanism due to minimizing wasteful operations. Sensor nodes in the proposed scheme start dying at later rounds as compared to other mentioned techniques due to limitation of control packet transmission and reception between sensor nodes over and over after every round. This help in saving energy of sensor nodes and thus prolonging their lifetime.

The overhead of the proposed algorithm is that when the batteries of sensor nodes reach to final stage, the graph of alive nodes falls sharply, as can be seen in Figure 4. The reason behind this is that nodes assigned as forwarders in the proposed algorithm do not change their state until their residual energy falls from a certain limit which is comparatively much larger than that of HHCA and other protocols, as explained in Section 4. Once all node batteries in the cluster reach the final stage, they start dying out frequently.

**Figure 5:**
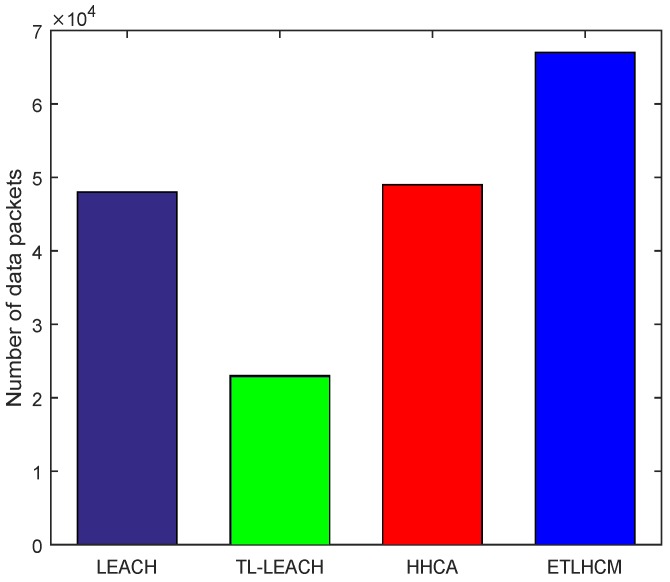
Total number of received data packets at base station.

In Figure 5, the total number of data packets transmitted and successfully received at base station are shown. The proposed scheme appears to have better result from HHCA, TL-LEACH, and LEACH protocols due to longer network lifetime. The good amount of energy that is used in other schemes during selection of CHs is saved to a large extent in the proposed scheme, and thus nodes remain alive for longer periods of time. This results directly in increasing the total number of successfully delivered data packets from sensor nodes to the base station. As many nodes die out in the network, that probability of data packet drop increases. In the proposed mechanism, sensor nodes remain alive for a longer time and thus the ratio of data packet drop is reduced.

**Figure 6:**
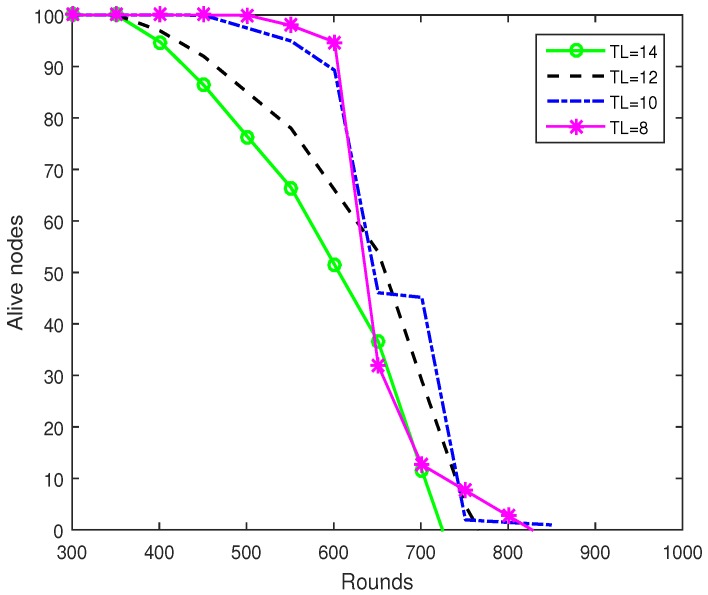
Network lifetime of proposed mechanism with different energy Total Level (TL).

Figure 6 shows network lifetime of the proposed scheme with different total energy levels TL, which is defined in Equation (6). As explained in Section 4, higher value of TL results in more balanced energy consumption of sensor nodes in the network, but on the cost of higher energy consumption due to frequent control packet exchange. As shown in the Figure 6, the reason that we cannot set very low value to TL is due to its tradeoff with balance energy consumption between all deployed sensor nodes. Based upon the network parameters which are defined in Table 1, the proposed scheme outperforms with value of TL = 10.

**Figure 7:**
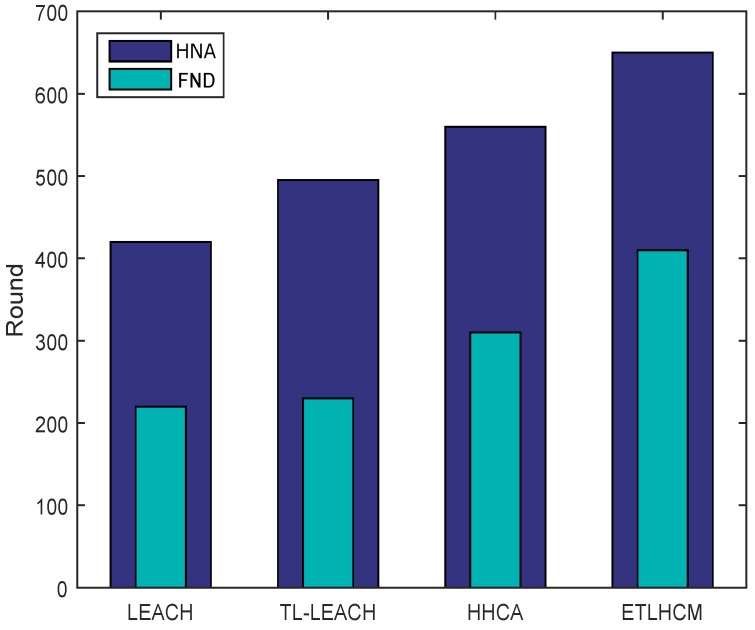
Network lifetime based on First Node Dies (FND) and Half of the Nodes Alive (HNA).

Figure 7 shows the network life of WSN based on two metrics called First Node Dies (FND) and Half of the Nodes Alive (HNA), which are proposed in [29]. FND metric shows the number of rounds in which the energy of first sensor node is exhausted and is considered dead. HNA metric on other hand shows number of rounds in which at least 50 percent of the deployed sensor nodes are functional. As WSN becomes almost useless after half of the deployed sensor nodes die out, we therefore focus more on HNA metric rather than Last Node Dies (LND) metric. HNA is also more useful metric in denser WSN as compared to FND metric, as network performance is not affected by dying out of single sensor node. As shown in Figure 7, the proposed scheme outperforms rest of techniques based on FND and HNA metrics.

## 6. Conclusions

Heavy control packet exchange between sensor nodes for obtaining an efficient forwarding route to the base station results in reducing overall network lifetime. This paper affirms that limiting wasteful operations in randomly deployed WSN are of paramount importance besides efficient selection of CHs in cluster based routing techniques. The wasteful operations are defined and reduced on the basis of energy consumption of all deployed sensor nodes. The proposed mechanism, evaluated in simple homogenous small scale network, limits wasteful operations in three-layer hybrid clustering routing protocol for WSN and results in 18 percent improvement in the network lifetime as compared to HHCA. In the future, we aim to implement the proposed mechanism in some application specific heterogeneous networks, and also to design and test it for large scale networks.

## Figures and Tables

**Table 1:** Simulation parameters and their values.

Parameters	Values
Network area	100 m × 100 m
Initial node energy	2 J
Number of sensor nodes	100
Data packet size	500 bytes
Packet header size	20 bytes
Control packet size	8 bytes
Bandwidth	1 Mbps
$E_{elc}$	50 nJ/bit
$\varepsilon_{fs}$	10 pJ/bit/m${}^{2}$
$\varepsilon_{mp}$	0.0013 pJ/bit/m${}^{4}$

## References

[1] Akyildiz I.F. (2002). Wireless sensor networks: A survey. Comput. Netw..

[2] Sohrabi K. (2000). Protocols for self-organization of a wireless sensor network. IEEE Pers. Commun..

[3] Heinzelman W.B., Chandrakasan Anantha P., Balakrishnan H. (2002). An application-specific protocol architecture for wireless microsensor networks. IEEE Trans. Wirel. Commun..

[4] Xiangning F., Song Y. Improvement on LEACH protocol of wireless sensor network. Proceedings of the International Conference on Sensor Technologies and Applications, 2007. SensorComm 2007.

[5] Hu J., Yuhui J., Liang D. A time-based cluster-head selection algorithm for LEACH. Proceedings of the IEEE Symposium on Computers and Communications, ISCC 2008.

[6] Xu J. Improvement of LEACH protocol for WSN. Proceedings of the 2012 9th International Conference on Fuzzy Systems and Knowledge Discovery (FSKD).

[7] Chunyao F. (2013). An energy balanced algorithm of LEACH protocol in WSN. Int. J. Comput. Sci. Issues (IJCSI).

[8] Lee J.-S., Kao T.-Y. (2016). An improved three-layer low-energy adaptive clustering hierarchy for wireless sensor networks. IEEE Internet Things J..

[9] Hoang D.C., Kumar R., Panda S.K. Fuzzy C-means clustering protocol for wireless sensor networks. Proceedings of the 2010 IEEE International Symposium on Industrial Electronics (ISIE).

[10] Ossama Y., Fahmy S. (2004). HEED: A hybrid, energy-efficient, distributed clustering approach for ad hoc sensor networks. IEEE Trans. Mob. Comput..

[11] Misra I., Dolui S.S., Das A. Enhanced energy-efficient adaptive clustering protocol for distributed sensor networks. Proceedings of the 2005 13th IEEE International Conference on Networks, 2005. Jointly Held with the 2005 IEEE 7th Malaysia International Conference on Communication.

[12] Youssef M.A., Mohamed F.Y., Khaled A.A. A constrained shortest-path energy-aware routing algorithm for wireless sensor networks. Proceedings of the Wireless Communications and Networking Conference, WCNC2002.

[13] Gautam N., Pyun J.Y. (2010). Distance aware intelligent clustering protocol for wireless sensor networks. J. Commun. Netw..

[14] Xufei M. (2011). Energy-efficient opportunistic routing in wireless sensor networks. IEEE Trans. Parallel Distrib. Syst..

[15] Mehdi T., Kavian Y.S., Siavoshi S. (2014). SEECH: Scalable energy efficient clustering hierarchy protocol in wireless sensor networks. IEEE Sens. J..

[16] Saman S. (2016). Geographical multi-layered energy-efficient clustering scheme for ad hoc distributed wireless sensor networks. IET Wirel. Sens. Syst..

[17] Aria N., Hunter T.E., Hedayat A. (2004). Cooperative communication in wireless networks. IEEE Commun. Mag..

[18] Ben N.A. A combined relay-selection and routing protocol for cooperative wireless sensor networks. Proceedings of the 2012 8th International Wireless Communications and Mobile Computing Conference (IWCMC).

[19] Natasha G. (2008). NOX: towards an operating system for networks. ACM SIGCOMM Comput. Commun. Rev..

[20] Jianping S. (2007). Centralized control of wireless sensor networks for real-time applications. IFAC Proc. Vol..

[21] Zhi L., Tsuda T., Watanabe H. Traffic deduction exploring sensor data’s intra-correlations in train track monitoring WSN. Proceedings of the 2015 IEEE SENSORS.

[22] Le N.P. (2017). Distributed hole-bypassing protocol in WSNs with constant stretch and load balancing. Comput. Netw..

[23] Habib M. (2018). Energy-efficient algorithm for reliable routing of wireless sensor networks. IEEE Trans. Ind. Electron..

[24] Zhi L. (2018). Data driven cyber-physical system for landslide detection. Mobile Networks and Applications.

[25] Mahmood J. (2018). Learning automaton based topology control protocol for extending wireless sensor networks lifetime. J. Netw. Comput. Appl..

[26] Habib M., Obaidat M.S. (2018). Learning automaton-based self-protection algorithm for wireless sensor networks. IET Netw..

[27] Estrin D. Wireless sensor networks tutorial part IV: Sensor network protocols. Proceedings of the MobiCom.

[28] Jin W.A.N.G. (2010). Hop-based energy aware routing algorithm for wireless sensor networks. IEICE Trans. Commun..

[29] Handy M.J., Haase M., Timmermann D. Low energy adaptive clustering hierarchy with deterministic cluster-head selection. Proceedings of the 4th International Workshop on Mobile and Wireless Communications Network.

